# The Effect of Different Promoters (La_2_O_3_, CeO_2_, and ZrO_2_) on the Catalytic Activity of the Modified Vermiculite-Based Bimetallic NiCu/EXVTM-SiO_2_ Catalyst in Methane Dry Reforming

**DOI:** 10.1021/acsomega.1c03959

**Published:** 2021-10-25

**Authors:** Zhaojun Meng, Zijun Wang

**Affiliations:** Key Laboratory for Green Processing of Chemical Engineering of Xinjiang Bingtuan, School of Chemistry and Chemical Engineering, Shihezi University, Beisi Road, Shihezi, Xinjiang 832003, China

## Abstract

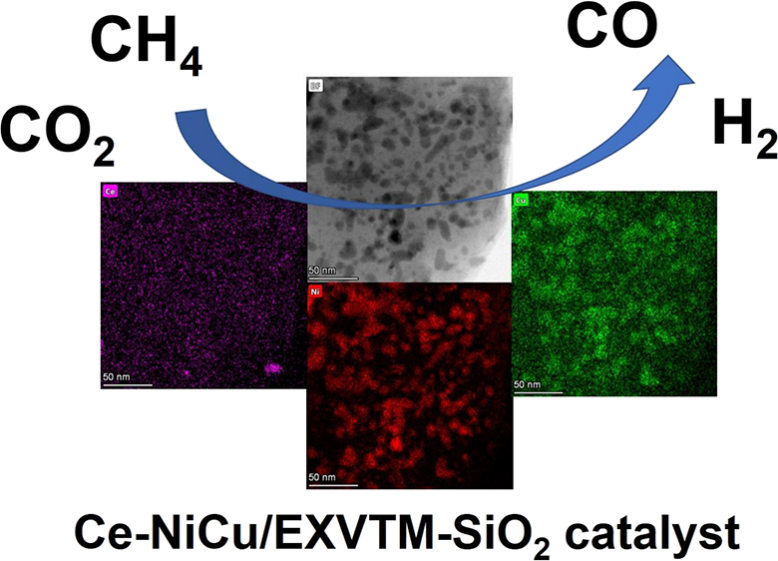

An X-NiCu/EXVTM-SiO_2_ (X = La, Ce, and Zr) catalyst was successfully prepared by using modified vermiculite as a support by the impregnation method. This experiment investigated the effects of La_2_O_3_, CeO_2_, and ZrO_2_ promoters on the activity of the NiCu/EXVTM-SiO_2_ catalyst. The study found that the addition of three different metal oxides did not improve the activity of the NiCu/EXVTM-SiO_2_ catalyst. On the contrary, some Ni active sites were covered by the promoter, which reduced the number of active sites, resulting in its catalytic activity lower than NiCu/EXVTM-SiO_2_. In addition, the promoted catalysts that were repeatedly calcined two times can significantly reduce the textural property as well as active sites of the catalyst, resulting in the lower activity. However, in X-NiCu/EXVTM-SiO_2_, Ce-NiCu/EXVTM-SiO_2_ showed relatively high initial catalytic activity, with the initial conversion rate of CH_4_ reaching 60.1% and the initial conversion rate of CO_2_ reaching 89.1%. This is mainly because the catalyst has a stronger basic site on the surface to facilitate the adsorption of CO_2_ molecules, and the smaller metal particle size is also conducive to the cleavage of C–H bonds.

## 1 Introduction

In the context of increasingly depleted fossil resources, global warming caused by excessive CO_2_ emissions, and serious environmental pollution, the shift to a low-carbon circular economy has become a global consensus,^1−4^ although mankind has made great efforts to find new energy and environmental protection. However, from the current point of view, there is still a certain gap between the actual situation and the realization of the goal of “carbon neutrality”. To deal with the greenhouse effect and meet the demand for clean and renewable energy, the dry reforming of methane has attracted much attention.^5,6^ In recent years, dry methane reforming (DRM) has become a research field with potential for development, because it consumes two main greenhouse gases (carbon dioxide and methane) and provides a way to convert them into valuable hydrocarbon products, which is undoubtedly an attractive environmental protection process.^7^ In addition, this reaction can convert high-CO_2_ and CH_4_ raw materials such as biogas and CO_2_-rich natural gas into high value-added synthesis gas (H_2_ and CO), which can be directly used as raw materials for the chemical industry.^8,9^

Nowadays, the use of precious metal (Rh, Ru, Pd, and Pt) and nonprecious metal (Ni, Co, and Fe) catalysts for dry reforming of methane has been studied.^5,10−14^ In high-temperature reactions, precious metal catalysts have attracted attention due to their excellent sintering resistance, higher activity, and stability. Among these precious metals, Ru and Rh show relatively high carbon deposition resistance and catalytic activity. Hou et al. studied the influence of different precious metals (Rh, Ru, Pt, Pd, and Ir) on alumina.^15^ The catalytic activity and stability trend of the catalyst is Rh/α-Al_2_O_3_ > Ru/α-Al_2_O_3_ > Ir/α-Al_2_O_3_ > Pd/α-Al_2_O_3_ > Pt/α-Al_2_O_3_. However, precious metal resources are scarce, the output is low, and the price is too expensive to be applied on an industrial scale. Therefore, domestic and foreign researchers mainly focus on the research of non-precious metal (Ni, Co, Cu, and Fe) catalysts, especially the supported Ni-based catalysts.^16^ However, DRM has two important limitations, mainly including high energy consumption due to the endothermic nature of the reaction and catalyst deactivation due to carbon deposition and metal sintering.^17^ To overcome the limitations of the catalyst itself, more and more scientists start from the structure of the catalytic support to study non-noble metal Ni-based catalysts that are resistant to high temperature, carbon deposition, and sintering. At present, researchers have been designing and constructing core–shell-type “nanoreactors”. Wang et al. started from the structure of a SiO_2_ support and synthesized a Ni@SiO_2_ catalyst with a small-size core–shell structure through the microemulsion method.^18^ It was found that the protective effect of silica nanospheres can limit the movement space of Ni nanoparticles, and the small size of Ni nanoparticles can reduce the carbon diffusion in Ni crystals. Therefore, the Ni@SiO_2_ catalyst exhibits excellent stability in the DRM reaction. Yang et al. reported the application of the Ni@SiO_2_ nanocatalyst with a yolk–shell structure in methane reforming.^19^ The study found that the Ni@SiO_2_ nanocatalyst with the yolk–shell structure can significantly reduce the sintering and carbon deposition of Ni particles. For the research of a core–shell catalyst, the most important is to use the confinement effect of the support itself. Therefore, the rational design of nickel-based catalysts with high activity and stability has become the key.

Limited to single-metal Ni-based catalysts, there are still defects in anti-carbon deposition and anti-sintering. Therefore, an effective way to improve the catalytic performance is to make Ni and another transition metal (such as Co, Fe, or Cu) form a bimetallic catalytic system. For methane reforming, bimetallic catalysts generally have higher reactivity and stability than single-metal catalysts. In recent years, Ni–Cu bimetallic systems with various supports and different synthesis methods have been studied. Song et al. studied the catalytic activity of hydrotalcite-derived Ni-Cu/Mg(Al)O catalysts in DRM reactions.^20^ It was found that the catalytic activity, stability, and anti-carbon deposition of the Ni-Cu/Mg(Al)O catalyst strongly depended on the composition of Ni–Cu. A higher Cu/Ni ratio (Cu/Ni = 1.0) will increase carbon deposition and promote rapid catalyst deactivation. The optimized Ni-Cu/Mg(Al)O (Cu/Ni = 0.25) catalyst exhibits stable catalytic performance even at temperatures as low as 723 K. Nataj et al. used Box–Behnken design to design Ni-Cu/Al_2_O_3_ catalysts with different Ni/Cu ratios for modeling and optimization of methane dry reforming.^21^ In this work, the addition of copper improved the reduction of the catalyst. In addition, the sintering of the active phase during the reaction is limited by the formation of Ni–Cu alloy, which leads to an increase in catalyst activity and stability. Nowadays, our research group has studied the expansion–acidification-modified vermiculite-supported NiM/EXVTM-SiO_2_ (M = Co, Cu, and Fe) bimetallic catalyst for methane dry reforming reaction.^22^ The study found that the NiCu/EXVTM-SiO_2_ catalyst showed relatively high catalytic activity and stability. However, carbon deposition causes the catalyst surface to be covered and hinders the activation of the reaction gas molecules, resulting in a decrease in catalytic activity. According to a previous report in the literature, both the acidic sites of the carrier and the large particles of nickel are likely to cause carbon deposits on the catalyst.^23^ In fact, in the DRM process, the reason for the formation of carbon deposits is affected by the ability of CO_2_ adsorption and activation. Under normal circumstances, reducing the Ni particle size and using a support with strong Lewis basicity can effectively reduce carbon deposition on the catalyst surface.^24,25^ In addition, increasing the oxygen storage capacity and redox performance can also promote the chemical adsorption of CO_2_ and facilitate the removal of carbon deposits.^26^ Most experimental studies have shown that promoters can enhance the thermal stability of the support and prevent the collapse of the support structure and the sintering of the active phase. At the same time, they also have the ability to improve the dispersibility of metals and increase the surface carbon of the gasification catalyst. As far as promoters are concerned, most of them are currently focused on the research of alkaline earth metal oxides and rare earth metal oxides.

Rare earth metal oxides as lattice defect promoters play an important role in the construction of anti-carbon deposition catalysts. CeO_2_ is known for its high ability to store and release oxygen, which can promote CO_2_ activation and inhibit carbon deposition.^27^ Zhang et al. successfully prepared a two-phase Ni–Ce catalyst on SBA-16 and studied the effect of cerium oxide on the structural stability and DRM performance of the NiCe/SBA-16 catalyst.^28^ The study found that compared with Ni/SBA-16 without adding cerium oxide, the strong interaction between Ni and CeO_2_ not only limits the growth of Ni but also reduces the close contact between the Ni metal and the SiO_2_ surface, thereby reducing the collapse of the SBA-16 skeleton caused by the reaction. In addition, compared with Ni/CeO_2_ and Ni/SBA-16, the NiCe/SBA-16 catalyst also shows ideal reducibility and stability in methane dry reforming. In addition to CeO_2_, La_2_O_3_ is also a commonly used rare earth promoter. Qian et al. studied the role of La in the Ni/SBA-15 catalyst in the methane reforming reaction.^29^ Studies have found that highly dispersed La effectively promotes the dissociation of CO_2_ into oxygen atoms, and the presence of La species enhances the metal dispersion of Ni on SBA-15 and improves the activity and stability of Ni/SBA-15 in dry reforming. Lanthanum oxide as an alkaline promoter can enhance the performance of the catalyst and improve the performance of carbon deposition resistance. In addition, La_2_O_3_ can react with CO_2_ on the catalyst surface and accelerate the conversion of CH_*x*_ surface substances.^30^ ZrO_2_ with high thermal stability also has certain alkaline and redox properties and is now mostly used as a support or promoter.^31^ Therdthianwong et al. studied the effect of zirconium oxide as a promoter of the Ni/Al_2_O_3_ catalyst on the methane reforming reaction.^32^ The study found that the addition of ZrO_2_ greatly improved the stability of the Ni/Al_2_O_3_ catalyst. At the same time, the active oxygen in zirconia can react with the carbon species on the surface of the catalyst, thereby inhibiting carbon deposition. It can be considered that the use of La_2_O_3_, CeO_2_, and ZrO_2_ as promoters can improve the reactivity and carbon deposition resistance of dry reforming.

Although CeO_2_, La_2_O_3_, and ZrO_2_ have been studied extensively as individual catalyst promoters of Ni catalysts for DRM, there are a few studies on bimetallic catalytic systems. Rare earth and alkaline promoter research on modified vermiculite is rarely reported. Therefore, it is imperative to study the effect of alkaline promoters on the catalytic activity of the modified vermiculite-based bimetallic NiCu/EXVTM-SiO_2_.

In this paper, an X-NiCu/EXVTM-SiO_2_ (X = La, Ce, and Zr) catalyst was successfully prepared by using modified vermiculite as a support by the impregnation method. The focus is on the effects of adding different metal oxides (La_2_O_3_, CeO_2_, and ZrO_2_) on the structure properties, catalytic activity, and stability of NiCu/EXVTM-SiO_2_ catalysts.

## 2 Results and Discussion

### 2.1 XRD Analysis

**Figure 1:**
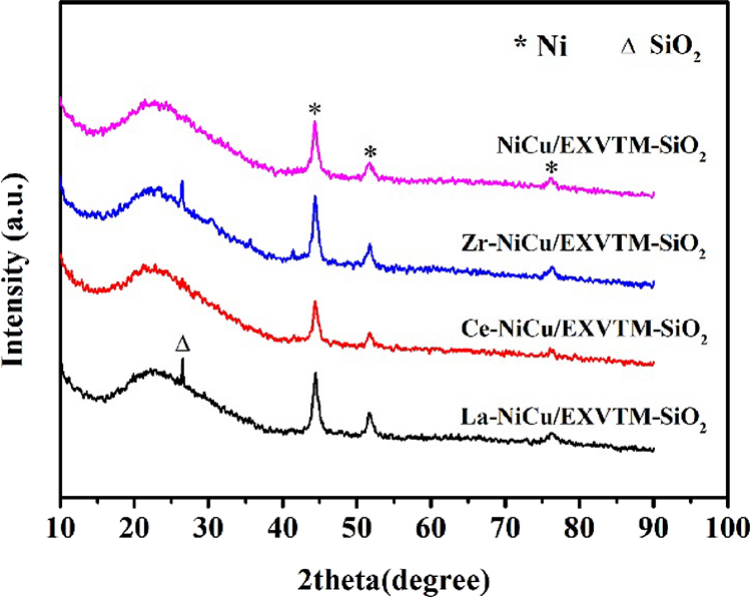
XRD patterns of X-NiCu/EXVTM-SiO_2_ (X = La, Ce, and Zr) reduced catalysts.

The X-ray diffraction (XRD) patterns of the X-NiCu/EXVTM-SiO_2_ and NiCu/EXVTM-SiO_2_ catalysts are shown in Figure 1. After four different catalysts are reduced, the characteristic peaks of Ni (no. 65-380) at 44.51°, 51.85°, and 76.37° in 2θ can be clearly observed, which correspond to the (111), (200), and (220) crystal planes of Ni (no. 65-380), respectively. It can be seen that the NiO in the catalyst has all been reduced to Ni after being reduced. It can be observed from the figure that there are differences in the intensity of Ni diffraction peaks. Compared with NiCu/EXVTM-SiO_2_, the Ni diffraction peak in the Ce-NiCu/EXVTM-SiO_2_ catalyst is broad and weak, which indicates that the active component Ni in the catalyst has a relatively small particle size and good dispersibility. This is mainly because the addition of CeO_2_ promoters promotes the dispersion of active metal Ni to a certain extent. Generally speaking, the reduction temperature of CuO is much lower than that of NiO, and Cu should be reduced first. However, the reduction peak of Cu was not detected in XRD, which indicated that Cu was highly dispersed in the support after reduction. At the same time, the characteristic diffraction peaks of La_2_O_3_, CeO_2_, and ZrO_2_ were also not detected, which may be related to the low loading of promoters.

### 2.2 BET Analysis

**Figure 2:**
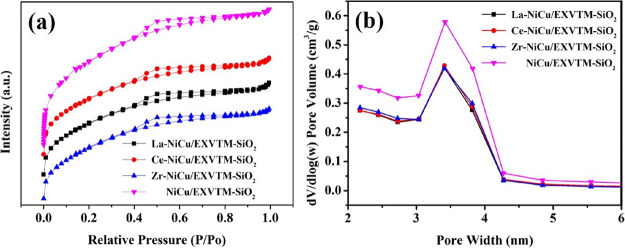
X-NiCu/EXVTM-SiO_2_ (X = La, Ce, and Zr) reduced catalysts: (a) N_2_ adsorption–desorption curves and (b) pore size distribution curves.

The N_2_ adsorption/desorption isotherm and pore size distribution curve of the reduction catalyst are shown in Figure 2. Based on Figure 2a, according to the IUPAC classification, the N_2_ adsorption and desorption curves of the X-NiCu/EXVTM-SiO_2_ (X = La, Ce, and Zr) and NiCu/EXVTM-SiO_2_ catalysts are all shown as typical IV-type curves with H_2_ hysteresis loops, which reveals the existence of a mesoporous structure in the four different catalyst samples. However, this pore structure can well anchor the active metal and has certain advantages in enhancing the anti-sintering performance of the active metal. At the same time, the existence of a mesoporous structure is also conducive to improving the mass transfer between gas-phase reactions.

In Figure 2b, the four catalysts showed similar pore size distribution curves, and the pore size distribution was in the range of 3–4 nm. As shown in Table 1, the pore sizes of La-NiCu/EXVTM-SiO_2_, Ce-NiCu/EXVTM-SiO_2_, Zr-NiCu/EXVTM-SiO_2_, and NiCu/EXVTM-SiO_2_ catalysts were 3.27, 3.27, 3.18, and 3.13 nm, respectively; the pore volumes were 0.101, 0.102, 0.100, and 0.148 cm^3^/g, respectively. In addition, the specific surface areas of La-NiCu/EXVTM-SiO_2_, Ce-NiCu/EXVTM-SiO_2_, Zr-NiCu/EXVTM-SiO_2_, and NiCu/EXVTM-SiO_2_ were 248.7, 253.7, 249.7, and 354.3 m^2^/g, respectively. However, after a 6 h stability test, the specific surface areas of the four catalysts decreased to 162.2, 168.3, 158.2, and 198.6 m^2^/g, respectively. This is mainly due to carbon deposition on the surface of the catalyst. In comparison, the BET results of the unpromoted catalyst (NiCu/EXVTM-SiO_2_) still have a relatively high specific surface area before and after the reaction. This indicates that the physical structural properties of the catalysts may be the main factors affecting the catalytic activity.

**Table 1:** Structural Properties of X-NiCu/EXVTM-SiO_2_ Reduced Catalysts

	surface area^a^ (m^2^/g)					actual loading^d^ (wt %)	
catalyst	reduced	spent	pore volume^b^ (cm^3^/g)	pore size^b^ (nm)	X-NiCu/EXVTM-SiO_2_ size^c^ (nm)	Ni	Cu
La-NiCu/EXVTM-SiO_2_	248.7	162.2	0.101	3.27	10.6	10.1	2.25
Ce-NiCu/EXVTM-SiO_2_	253.7	168.3	0.102	3.27	7.4	10.6	2.46
Zr-NiCu/EXVTM-SiO_2_	249.7	158.2	0.100	3.18	10.5	10.1	2.27
NiCu/EXVTM-SiO_2_	354.3	198.6	0.148	3.13	9.1	10.4	2.34

a Obtained from the BET method.

b The pore volume and pore diameter were obtained with the BJH approach.

c Calculation from TEM particle size data.

d Measured by ICP-OES.

### 2.3 H_2_-TPR Analysis

**Figure 3:**
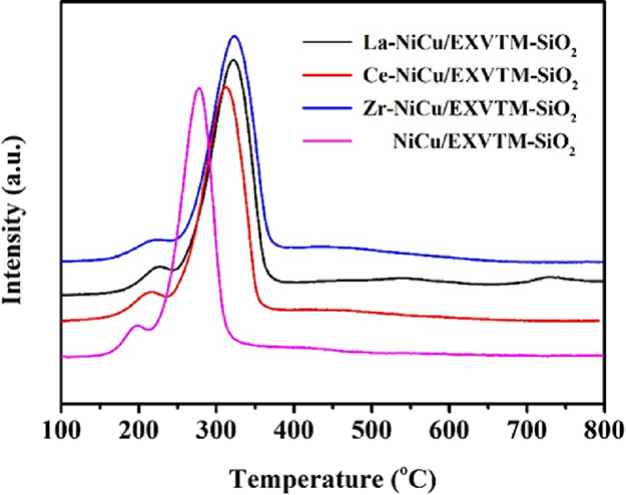
H_2_-TPR curves of X-NiCu/EXVTM-SiO_2_ fresh catalysts.

H_2_-TPR is used to determine the reduction behavior of X-NiCu/EXVTM-SiO_2_ catalysts. The reduction curve of X-NiCu/EXVTM-SiO_2_ catalysts is shown in Figure 3. To compare the effects of La_2_O_3_, CeO_2_, and ZrO_2_ promoters on the reducibility of the NiCu/EXVTM-SiO_2_ catalyst, this experiment also measured the TPR curve of the NiCu/EXVTM-SiO_2_ catalyst. As shown in Figure 3, all the catalysts showed two different reduction peaks. The low-temperature reduction peak is attributed to the reduction of CuO, and the high-temperature reduction peak is attributed to the reduction of Ni–Cu alloy or highly dispersed NiO. Compared with the NiCu/EXVTM-SiO_2_ catalyst, the reduction temperature of the X-NiCu/EXVTM-SiO_2_ catalyst is increased after adding promoters.

However, the reduction behavior of the X-NiCu/EXVTM-SiO_2_ (X = La, Ce, and Zr) catalyst is also different. The Ce-NiCu/EXVTM-SiO_2_ catalyst has a relatively low reduction temperature, indicating that the interaction between the support and the active components is weak. La-NiCu/EXVTM-SiO_2_ and Zr-NiCu/EXVTM-SiO_2_ catalysts have relatively high reduction temperatures, indicating that the interaction between the active metal and the support is strong. However, this strong interaction seems to be not conducive to improving the reaction activity of the catalyst in the reforming reaction.^33^

### 2.4 CO_2_-TPD Analysis

**Figure 4:**
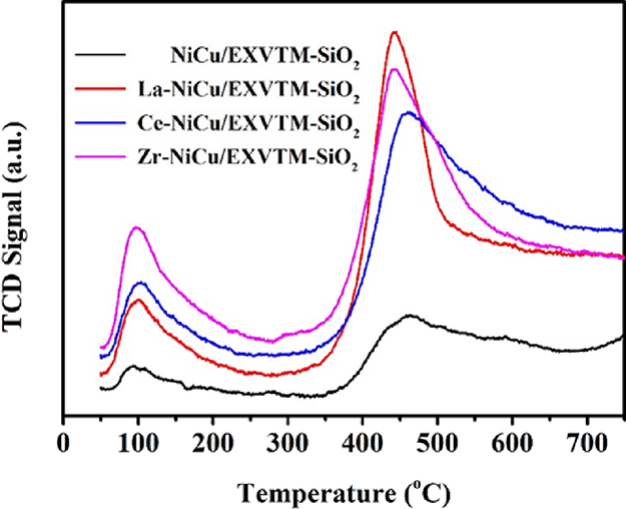
CO_2_-TPD curves of X-NiCu/EXVTM-SiO_2_ reduced catalysts.

CO_2_-TPD is a characterization method used to study the basic active sites on the catalyst surface. The CO_2_-TPD curve of the reduced X-NiCu/EXVTM-SiO_2_ catalyst is shown in Figure 4. All catalysts showed two CO_2_ desorption peaks, indicating that there are two different active sites for CO_2_ adsorption on the catalyst surface, which corresponds to the two adsorption centers with stronger basicity and weaker basicity, respectively. The low-temperature desorption peak can be attributed to physical adsorption, and the high-temperature desorption peak can be attributed to chemical adsorption. Therefore, the desorption peak that appears at the peak temperature around 100 °C can be attributed to the removal of physical adsorption and weak chemical adsorption of CO_2_ molecules; the desorption peak that appears at the peak temperature around 400–500 °C can be attributed to the removal of strong chemically adsorbed CO_2_ molecules. The addition of La_2_O_3_, CeO_2_, and ZrO_2_ promoters increases the desorption temperature of the NiCu/EXVTM-SiO_2_ catalyst. In the CO_2_-TPD spectrum, the desorption temperature of the X-NiCu/EXVTM-SiO_2_ catalyst is also different. Among all the catalysts, the NiCu/EXVTM-SiO_2_ catalyst has the lowest desorption temperature, indicating that the NiCu/EXVTM-SiO_2_ catalyst has weak Lewis basicity and weaker CO_2_ adsorption. The Ce-NiCu/EXVTM-SiO_2_ catalyst has the highest desorption temperature, indicating that the Ce-NiCu/EXVTM-SiO_2_ catalyst has strong Lewis basicity and exhibits strong adsorption of CO_2_.

### 2.5 TEM and EDX Mapping Analysis

**Figure 5:**
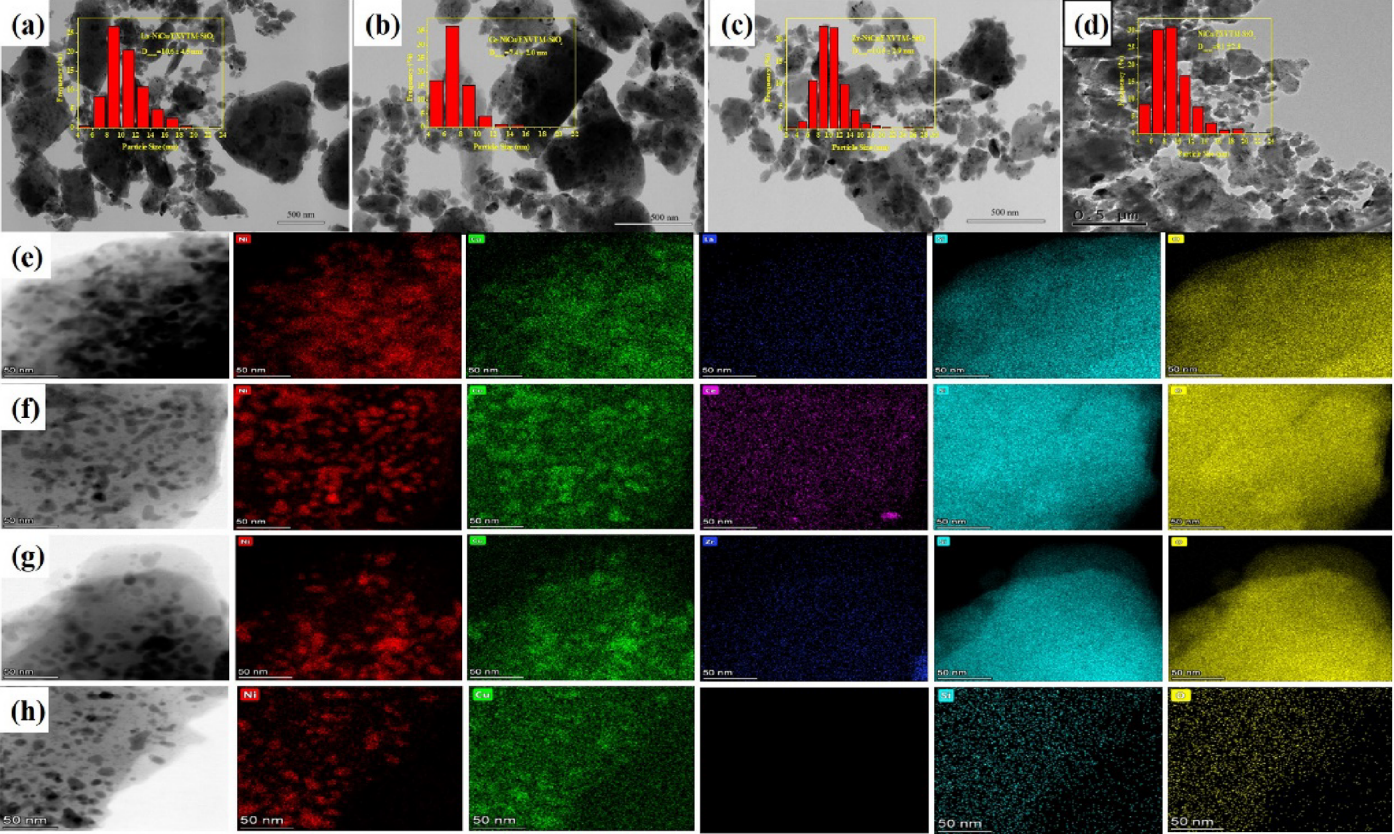
TEM and EDX mapping images of reduced catalyst samples: (a, e) La-NiCu/EXVTM-SiO_2_, (b, f) Ce-NiCu/EXVTM-SiO_2_, (c, g) Zr-NiCu/EXVTM-SiO_2_, and (d, h) NiCu/EXVTM-SiO_2_.

The TEM images of La-NiCu/EXVTM-SiO_2_, Ce-NiCu/EXVTM-SiO_2_, Zr-NiCu/EXVTM-SiO_2_, and NiCu/EXVTM-SiO_2_ catalysts are shown in Figure 5a–d, respectively. Fine particles can be clearly observed from the TEM image, indicating that the active component has been successfully loaded onto the support. By counting the particle size of nanoparticles in the transmission electron microscopy (TEM) image, the particle size distribution histogram shown in the TEM inset is obtained. As shown in the illustration, the average particle size of La-NiCu/EXVTM-SiO_2_ is 10.6 nm, the average particle size of Ce-NiCu/EXVTM-SiO_2_ is 7.4 nm, the average particle size of Zr-NiCu/EXVTM-SiO_2_ is 10.5 nm, and the average particle size of NiCu/EXVTM-SiO_2_ is 9.1 nm. In comparison, Ce-NiCu/EXVTM-SiO_2_ has a smaller particle size and the active components in the catalyst have better dispersibility. Related studies have shown that the level of catalytic activity is related to the size of Ni particles.^34^ The smaller the Ni particle size, the higher its catalytic activity. Therefore, the catalytic activity of the Ce-NiCu/EXVTM-SiO_2_ catalyst should be better than those of La-NiCu/EXVTM-SiO_2_, Zr-NiCu/EXVTM-SiO_2_, and NiCu/EXVTM-SiO_2_ catalysts.

EDX mapping further determined the microstructure and element distribution of the nanocatalyst. Figure 5e–h respectively shows the EDX elemental mapping of La-NiCu/EXVTM-SiO_2_, Ce-NiCu/EXVTM-SiO_2_, Zr-NiCu/EXVTM-SiO_2_, and NiCu/EXVTM-SiO_2_ catalysts. It can be clearly seen from the EDX mapping picture that various elements of the X-NiCu/EXVTM-SiO_2_ and NiCu/EXVTM-SiO_2_ catalysts are dispersed on the surface of the support. The map of Ni, Cu, Si, and O elements reflects the distribution state of the main elements in the catalyst, and La, Ce, and Zr also clearly show the dispersion state of the promoter elements. From the mapping diagrams of the four catalysts, it is found that the dispersion of Ni and Cu in the Ce-NiCu/EXVTM-SiO_2_ catalyst is better. The addition of CeO_2_ promoters promoted the dispersion of metallic Ni and Cu to a certain extent. At the same time, active metals also promote the dispersibility of CeO_2_. This shows that the active metals and promoters have played a mutually promoting role. However, in the Ce-NiCu/EXVTM-SiO_2_ catalyst, the mapping area between CeO_2_ and the active metal (Ni and Cu) overlaps with each other, which results in the reduction of active sites of the catalyst, resulting in its catalytic activity lower than that of NiCu/EXVTM-SiO_2_.

### 2.6 Catalytic Activity Analysis

**Figure 6:**
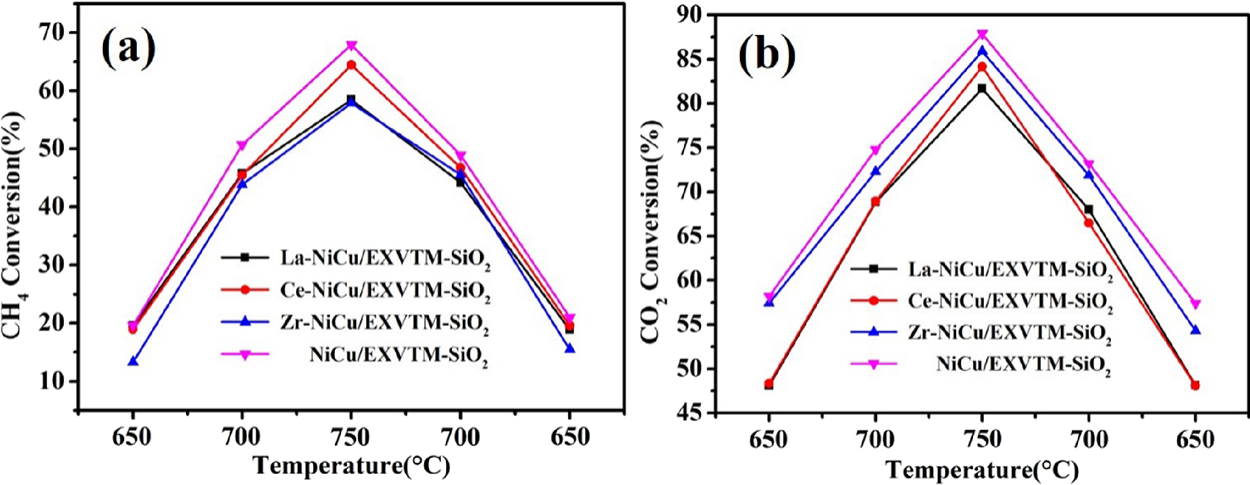
X-NiCu/EXVTM-SiO_2_ (X = La, Ce, and Zr) catalyst conversion curves of (a) CH_4_ and (b) CO_2_ at different reaction temperatures.

Figure 6 reflects the change trend of the catalytic activity of the X-NiCu/EXVTM-SiO_2_ and NiCu/EXVTM-SiO_2_ catalysts in the range of 650–750 °C with temperature. As the reaction temperature increases, the catalytic activity of the X-NiCu/EXVTM-SiO_2_ (X = La, Ce, and Zr) and NiCu/EXVTM-SiO_2_ catalysts gradually increases. This is mainly because the methane dry reforming reaction is a strongly endothermic reaction (CH_4_ + CO_2_ ⃗ 2H_2_ + 2CO, Δ*H* = +246 kJ/mol), and a high temperature is conducive to the progress of the catalytic reaction. At a temperature of 750 °C, the Ce-NiCu/EXVTM-SiO_2_ catalyst showed a relatively high conversion rate of CH_4_ and CO_2_, the conversion rates of which were 64.4 and 84.2%, respectively. However, the catalytic activity of NiCu/EXVTM-SiO_2_ is always higher than that of the X-NiCu/EXVTM-SiO_2_ catalyst in the whole reaction process. This is mainly because the NiCu/EXVTM-SiO_2_ catalyst has a relatively high specific surface area and active sites.

For the four different catalysts, the conversion rates of CH_4_ and CO_2_ have the same changing trend. Moreover, in the entire temperature range, the conversion rate of CO_2_ is always higher than that of CH_4_, which is mainly due to the existence of water-gas shift reaction and Boudouard reaction in this reaction.^35^

### 2.7 Catalytic Performance

**Figure 7:**
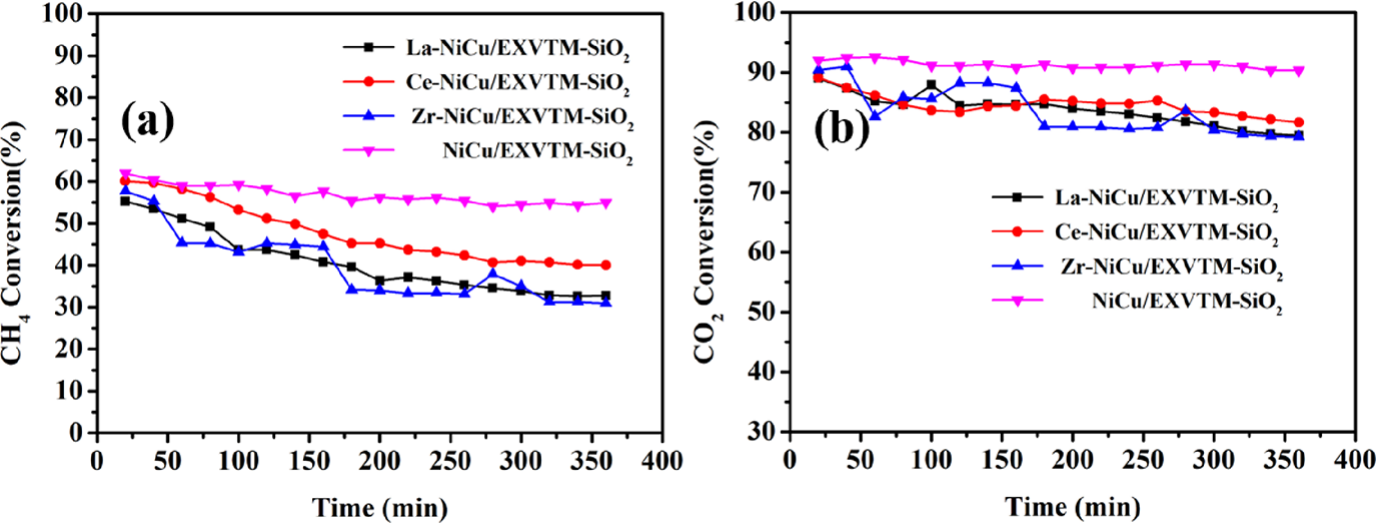
X-NiCu/EXVTM-SiO_2_ catalysts for (a) CH_4_ conversion and (b) CO_2_ conversion for DRM reaction. Reaction conditions: *P* = 1 atm, *T* = 750 °C, GHSV = 18,000 mL/(g·h), *W*_cat_ = 0.12 g, CO_2_:CH_4_:N_2_ = 1.3:1.3:1.

According to the relationship between catalytic activity and temperature change, the four catalysts all have the highest conversion rate at 750 °C. Therefore, the stability test of the catalyst was carried out at 750 °C. Figure 7 shows the stability test of the X-NiCu/EXVTM-SiO_2_ (X = La, Ce, and Zr) catalysts at a temperature of 750 °C and a space velocity of 18,000 mL/(g·h). To study the influence of different promoters on the catalytic activity, the experiment also measured the stability of the NiCu/EXVTM-SiO_2_ catalyst.

It can be seen from Figure 7 that the initial conversion rates of CH_4_ for La-NiCu/EXVTM-SiO_2_, Ce-NiCu/EXVTM-SiO_2_, and Zr-NiCu/EXVTM-SiO_2_ catalysts were 55.2, 60.1, and 57.7%; the initial conversion rates of CO_2_ were 89.0, 89.1, and 90.4%, respectively. The initial catalytic activity of the three catalysts is different, and the Ce-NiCu/EXVTM-SiO_2_ catalyst shows higher catalytic activity. This is mainly due to the better dispersion of active components and smaller particle size in the Ce-NiCu/EXVTM-SiO_2_ catalyst, which makes the catalyst have a stronger ability to break a C–H bond and thus show higher catalytic activity. With the progress of the catalytic reaction, the conversion rates of CH_4_ and CO_2_ in the X-NiCu/EXVTM-SiO_2_ (X = La, Ce, and Zr) catalysts have decreased. This is mainly caused by carbon deposits and active metal sintering. It is worth noting that the catalytic activity of the NiCu/EXVTM-SiO_2_ catalyst does not change much as the catalytic reaction progresses. The addition of promoters did not improve the catalytic activity of the NiCu/EXVTM-SiO_2_ catalyst. On the contrary, the addition of La_2_O_3_, CeO_2_, and ZrO_2_ promoters covered part of the active sites in the NiCu/EXVTM-SiO_2_ catalyst, which resulted in the catalytic activity of X-NiCu/EXVTM-SiO_2_ being lower than that of the NiCu/EXVTM-SiO_2_ catalyst. This is consistent with the results of EDX mapping analysis. In addition, the promoted catalysts that were repeatedly calcined two times can significantly reduce the textural property and active sites of the catalyst, resulting in the lower activity.

### 2.8 TG-DSC Analysis

**Figure 8:**
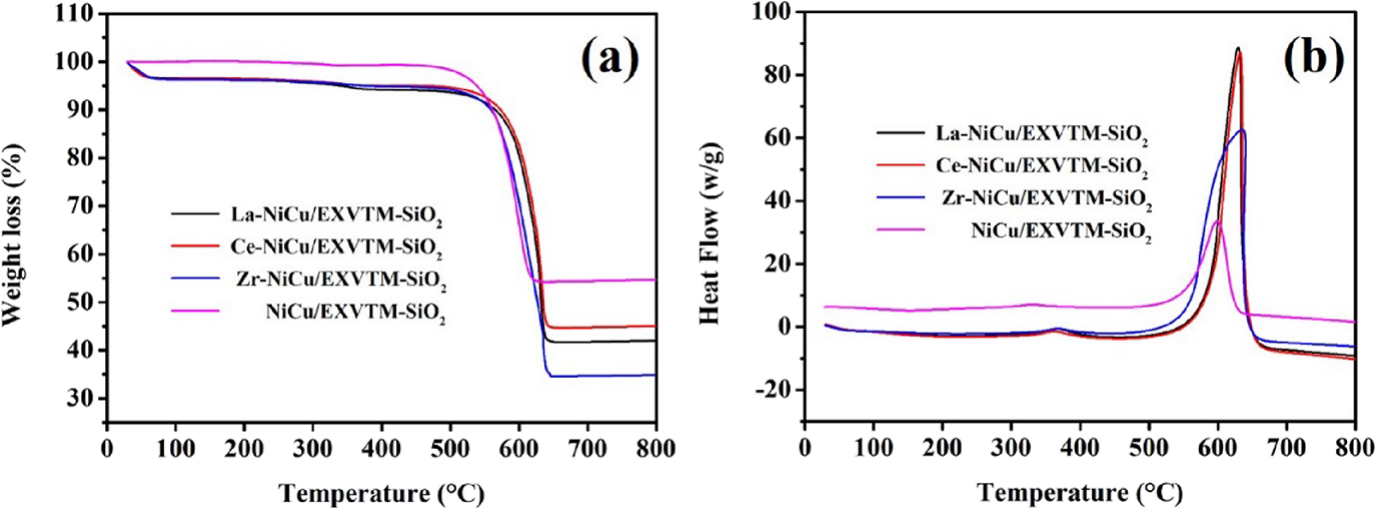
TG-DSC curves of X-NiCu/EXVTM-SiO_2_ spent catalysts: (a) TG curves and (b) DSC curves.

Figure 8 shows the TG-DSC curves of the X-NiCu/EXVTM-SiO_2_ and NiCu/EXVTM-SiO_2_ spent catalysts. The thermogravimetric (TG) curve measures the amount of carbon deposited on the catalyst, while the DSC curve studies the endothermic and exothermic effects of the sample. It can be observed from the figure that the TG-DSC curves of the X-NiCu/EXVTM-SiO_2_ and NiCu/EXVTM-SiO_2_ catalysts can be divided into three stages. For the first stage, in the range of 0–100 °C, a small amount of weight loss appears on the TG curve, which can be attributed to the removal of trace water and gas impurities on the catalyst surface. For the second stage, in the range of 300–400 °C, a small exothermic peak appears in the DSC curve, which can be attributed to the oxidation of the active metal in the catalyst, which corresponds to the slight increase in weight in the TG curve. For the third stage, in the range of 500–700 °C, the TG curve has obvious weight loss, which can be attributed to the oxidation of carbon deposition on the catalyst surface to CO and CO_2_. This corresponds to the strong exothermic signal in the DSC curve. In Figure 8, you can clearly see that the carbon deposit of the NiCu/EXVTM-SiO_2_ catalyst is significantly lower than those of the X-NiCu/EXVTM-SiO_2_ (X = La, Ce, and Zr) catalysts. This is mainly because the promoted catalysts that were repeatedly calcined two times can significantly reduce the textural property as well as active sites of the catalyst, resulting in the lower activity and higher carbon deposition. In addition, the weight loss trend is Zr-NiCu/EXVTM-SiO_2_ > La-NiCu/EXVTM-SiO_2_ > Ce-NiCu/EXVTM-SiO_2_. This shows that among the three different catalysts after the same reaction time, the Ce-NiCu/EXVTM-SiO_2_ catalyst has less carbon deposits. On the one hand, the reason can be attributed to the fact that the lattice oxygen that exists in CeO_2_ can play a role in oxidizing the surface carbon of the catalyst. On the other hand, CeO_2_ has strong Lewis basicity, which exhibits strong adsorption of CO_2_ and can promote the conversion of carbon (C + CO_2_ ⃗ 2CO) on the catalyst surface under high temperature conditions.

## 3 Conclusions

(1) The addition of La_2_O_3_, CeO_2_, and ZrO_2_ did not improve the catalytic activity of NiCu/EXVTM-SiO_2_. On the one hand, the promoters covered part of the active sites in the NiCu/EXVTM-SiO_2_ catalyst, which resulted in the catalytic activity of X-NiCu/EXVTM-SiO_2_ being lower than that of the NiCu/EXVTM-SiO_2_ catalyst. On the other hand, the promoted catalysts that were repeatedly calcined two times can significantly reduce the textural property as well as active sites of the catalyst, resulting in the lower activity. However, among the NiCu/EXVTM-SiO_2_ catalysts modified with three different promoters, the Ce-NiCu/EXVTM-SiO_2_ catalyst showed relatively high catalytic activity. This is because the particle size of the active components in the catalyst is smaller and the dispersibility is better.
(2) As the reforming reaction progresses, carbon deposits on the surface of the X-NiCu/EXVTM-SiO_2_ catalyst block the gas transmission channel and cover the active sites, which reduces the stability of the catalyst. However, the carbon deposition amount of Ce-NiCu/EXVTM-SiO_2_ is significantly lower than those of La-NiCu/EXVTM-SiO_2_ and Zr-NiCu/EXVTM-SiO_2_. This is mainly because the lattice oxygen in CeO_2_ itself and the strong Lewis basicity of the Ce-NiCu/EXVTM-SiO_2_ catalyst all contribute to the conversion of carbon deposits.

## 4 Experimental Section

### 4.1 Catalyst Preparation

#### 4.1.1 Preparation of the Support (EXVTM-SiO_2_)

The preparation method of EXVTM-SiO_2_ (i.e., hierarchically layered porous SiO_2_) was described in our previous work.^22^

#### 4.1.2 Preparation of the NiCu/EXVTM-SiO_2_ Catalyst

A NiCu/EXVTM-SiO_2_ bimetallic catalyst with a Ni:Cu molar ratio of 10:3 and a Ni loading of 10 wt % was prepared by the impregnation method. A certain amount of Cu(NO_3_)_2_·*x*H_2_O as the metal precursor was weighed and dissolved in an appropriate amount of deionized water. A metered amount of Ni(NO_3_)_2_·6H_2_O was dissolved in a precursor solution of copper nitrate, after which a certain amount of EXVTM-SiO_2_ support was added overnight. Afterward, the samples were dried by heating at 120 °C for 12 h in an oven and finally calcined in a muffle furnace at 750 °C for 4 h.

#### 4.1.3 Preparation of the X-NiCu/EXVTM-SiO_2_ (X = La, Ce, and Zr) Catalysts

The same impregnation method was used to prepare the X-NiCu/EXVTM-SiO_2_ catalysts with 2% La_2_O_3_, CeO_2_, and ZrO_2_ mass fraction (La_2_O_3_, CeO_2_, and ZrO_2_ respectively accounted for the percentage of the modified support mass). The detailed steps were as follows: A certain amount of La(NO_3_)_3_·6H_2_O, Ce(NO_3_)_3_·6H_2_O, and Zr(NO_3_)_4_·5H_2_O was weighed, dissolved into 10 mL of deionized water, and then respectively immersed in a metered amount of NiCu/EXVTM-SiO_2_ fresh catalyst. After standing for 12 h, it was placed in an oven at 120 °C, heated for 12 h, and then placed in a muffle furnace at 550 °C for 4 h. To obtain the reduced catalyst, the calcined catalyst needed to be reduced at a temperature of 700 °C and 50 mL/min H_2_-Ar atmosphere for 2 h to finally obtain the X-NiCu/EXVTM-SiO_2_ (X = La, Ce, and Zr) catalyst.

### 4.2 Catalyst Characterization Techniques

The crystal phase structure of the catalyst was characterized by a D8-Advance X-ray diffractometer produced by Bruker, Germany. The specific surface area and mesoporous structure of the catalyst were tested using the Micromeritics Instrument ASAP 2460 automatic rapid specific surface and porosity analyzer produced by Micromeritics Instrument. The temperature-programmed reduction (H_2_-TPR) adopted the automated chemisorption analyzer produced by Quantachrome Instruments (Chem Star, USA) to determine the reduction temperature of the catalyst. Determination of basic active sites on the catalyst surface by the CO_2_-TPD (AutoChem II2920) was carried out by Micromeritics Instrument. The microstructure and element distribution of the catalyst were observed by the TALOS F200 transmission electron microscope produced by FEI Company in the United States. The carbon deposit of the catalyst was measured by the DSC/TGA (SDT, Q600) type thermogravimetric analyzer produced by TA Instruments.

### 4.3 Catalytic Activity and Stability Tests

Under normal pressure, a micro fixed-bed reactor was used to test the activity and stability of the catalyst. A total of 0.12 g of catalyst was weighed and placed in the middle of a reaction tube with an inner diameter of 10 mm and a length of 460 mm. Before the feed gas (CH_4_ and CO_2_) was introduced into the reactor, the catalyst was reduced in situ at 700 °C for 2 h with 50 mL/min 10% H_2_-Ar mixed gas. Then, the catalytic activity was tested in the temperature range of 650–750 °C (with an interval of 50 °C) under the condition of space velocity of 18,000 mL/(g·h) at atmospheric pressure. The stability test experimental conditions were as follows: *P* = 1 atm, *T* = 750 °C, CH_4_:CO_2_:N_2_ = 13:13:10 mL/min, and the space velocity was 18,000 mL/(g·h). On-line analysis of product components was carried out by gas chromatography (Fuli 9790).
